# Inhibition of long non-coding RNA NEAT1 impairs myeloid differentiation in acute promyelocytic leukemia cells

**DOI:** 10.1186/1471-2407-14-693

**Published:** 2014-09-23

**Authors:** Chengwu Zeng, Yan Xu, Ling Xu, Xibao Yu, Jingjing Cheng, Lijian Yang, Shaohua Chen, Yangqiu Li

**Affiliations:** Institute of Hematology, Medical College, Jinan University, Guangzhou, 510632 China; Key Laboratory for Regenerative Medicine of Ministry of Education, Jinan University, Guangzhou, 510632 China

## Abstract

**Background:**

Acute promyelocytic leukemia (APL) is characterized by the reciprocal translocation t(15;17), which fuses PML with retinoic acid receptor alpha (RARα). Although PML-RARα is crucially important for pathogenesis and responsiveness to treatment, the molecular and cellular mechanisms by which PML-RARα exerts its oncogenic potential have not been fully elucidated. Recent reports have suggested that long non-coding RNAs (lncRNAs) contribute to the precise control of gene expression and are involved in human diseases. Little is known about the role of lncRNA in APL.

**Methods:**

We analyzed NEAT1 expression in APL samples and cell lines by real-time quantitative reverse transcription-PCR (qRT-PCR). The expression of PML-RARα was measured by Western blot. Cell differentiation was assessed by measuring the surface CD11b antigen expression by flow cytometry analysis.

**Results:**

We found that nuclear enriched abundant transcript 1 (NEAT1), a lncRNA essential for the formation of nuclear body paraspeckles, is significantly repressed in de novo APL samples compared with those of healthy donors. We further provide evidence that NEAT1 expression was repressed by PML-RARα. Furthermore, significant NEAT1 upregulation was observed during all-trans retinoic acid (ATRA)-induced NB4 cell differentiation. Finally, we demonstrate the importance of NEAT1 in myeloid differentiation. We show that reduction of NEAT1 by small interfering RNA (siRNA) blocks ATRA-induced differentiation.

**Conclusions:**

Our results indicate that reduced expression of the nuclear long noncoding RNA NEAT1 may play a role in the myeloid differentiation of APL cells.

**Electronic supplementary material:**

The online version of this article (doi:10.1186/1471-2407-14-693) contains supplementary material, which is available to authorized users.

## Background

Acute promyelocytic leukemia (APL) is characterized by an aberrant chromosomal translocation that fuses a portion of the promyelocytic leukemia (*PML*) gene with the retinoic acid receptor α (*RARα*) gene [1], and subsequent expression of the PML-RARα oncoprotein causes a block at the promyelocytic differentiation stage. All-trans retinoic acid (ATRA) has been successfully used as a leukemia therapy to target the transcriptional repression mediated by the PML-RARα fusion protein. The treatment of the t(15;17) APL with ATRA induces the differentiation of t(15;17) blasts and causes disease regression [2, 3] presumably through degradation of the chimeric protein encoded by the *PML-RARα* oncogene [4–6].

Although PML-RARα is crucially important for pathogenesis and responsiveness to treatment, the mechanism by which PML-RARα exerts its oncogenic potential remains unclear. Previous studies have proposed that PML-RARα acts as a strong transcriptional repressor for target genes by directly binding their promoter regions, which are thought to include genes indispensable for myeloid differentiation and apoptosis [7, 8]. However, the number of identified PML-RARα target genes is limited [7]. Given the structural and functional complexity of PML-RARα, indirect effects of PML-RARα may play a significant role in leukemic transformation. Indeed, PML-RARα may even activate the expression a subset of genes [9, 10]. These data point to the necessity for addressing the issue of indirect PML-RARα-mediated gene expression control.

Mammalian transcriptome studies have revealed large numbers of long transcripts that have no protein-coding potential. We previously demonstrated that microRNAs play a significant role in the regulation of differentiation, proliferation and apoptosis [11, 12]. Compared with the research progress of microRNAs, there are thousands of longer transcripts whose functions are unknown. Recently, several long non-coding RNAs (lncRNAs) have been implicated in many types of cancers [13, 14]. Our preliminary data showed that NEAT1 is highly expressed in the APL cell line NB4. NEAT1 (nuclear paraspeckle assembly transcript 1) is a nuclear-restricted long non-coding RNA that has two isoforms: 3.7 kb NEAT1_1 and 23 kb NEAT1_2 [15, 16]. This non-coding RNA was recently revealed to be an architectural component of a subnuclear structure called the paraspeckle, which is suggested to be involved in regulating gene expression by retaining mRNAs for editing in the nucleus [16, 17]. Although considerable progress has been made into the paraspeckle composition, formation, and molecular organization, the biological function of paraspeckles and the role of the NEAT1 lncRNAs are incompletely defined. In addition, it is not yet clear whether lncRNAs are involved in APL pathogenesis. In this study, we aimed to characterize the role and regulation of NEAT1 in APL.

## Methods

### Patients and samples

A total of 43 peripheral blood samples including 31 APL samples at diagnosis and 12 normal donors with informed consent. All of the procedures were conducted according to the guidelines of the Medical Ethics Committees of the Health Bureau of the Guangdong Province of China, and ethical approval was obtained from the Ethics Committee of Medical School of Jinan University for this study.

### Cell lines and cell cultures

NB4, NB4-R2 and U937-PR9 cell lines were kindly provided by Dr. Yueqin Chen (Sun Yat-sen University, Guangzhou, China) and cultured in RPMI 1640 containing 10% fetal bovine serum. U937-PR9 contains a zinc-inducible PML-RARα constructed from U937 [18]. The cells were cultured in a humidified atmosphere containing 5% CO_2_ at 37°C. ATRA was purchased from Sigma-Aldrich and used at the following final concentrations: 1 μM ATRA (stock 10 mM in EtOH).Cell differentiation was assessed by measuring the surface ITGAM/CD11b antigen expression by flow cytometry analysis.

### Quantitative real-time PCR analysis

qRT-PCR was performed to detect mature lncRNAs and mRNA expression. Briefly, RNA was reverse-transcribed to cDNA using High-Capacity cDNA Reverse Transcription Kits(Applied Biosystems). *ATCB* served as internal control. Primers were as follows: NEAT1 forward, 5′-CTTCCTCCCTTTAACTTATCCATTCAC-3′; NEAT1 reverse, 5′-CTCTTCCTCCACCATTACCAACAATAC-3′; NEAT1_2 forward, 5′- CAGTTAGT TTATCAGTTCTCCCATCCA-3′; NEAT1_2 reverse, 5′-GTTGTTGTCGTCACCTTTCAACTCT -3′. qRT-PCR cycling program: 95°C for 15 min, followed by 40 cycles at 95°C for 10 s and 60°C for 30 s.

### Transfection

NB4 cells were transfected using the Neon® Transfection System (Invitrogen) with 100 pmol of oligonucleotides in 10 μl reactions. Transfection was performed as described previously [19]. The sequences of small interfering RNA (siRNA) that specifically targets the breakpoint region of PML-RARα were designed as previously described [20]. The following siRNA sequences targeting the NEAT1 are as follows: 5′-GUGAGAAGUUGCUUAGAAACUUUCC-3′.

### Western blot

Cells (NB4 and U937-PR9) were washed twice in phosphate-buffered saline (PBS) and lysed on ice for 30 min in RIPA buffer. Protein extracts were separated in a sodium dodecyl sulfate polyacrylamide electrophoresis (SDS-PAGE) gel. The proteins were then transferred to a polyvinylidenedifluoride (PVDF) membrane and probed with anti-RARα (C-20; Santa Cruz Biotechnology) and anti-β-actin (Sigma-Aldrich) antibodies.

### Statistical analysis

Data were expressed as the mean ± SD of 3 independent experiments. The significance of the differences between groups was determined by a two-tailed Student t test. A P-value <0.05 was considered significant.

## Results

### NEAT1 express levels were downregulated in APL

**Figure 1:**
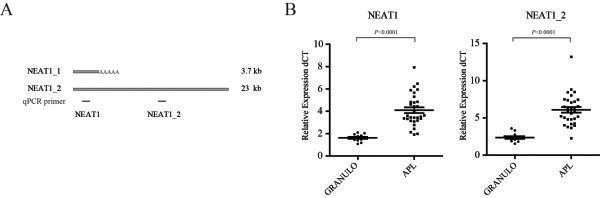
**The lncRNA NEAT1 is significantly down-regulated in APL primary patient samples. (A)** The NEAT1 isoforms are shown schematically. The black boxes indicate the position of sequences amplified by qRT-PCR. **(B)** Comparison of NEAT1 expression in granulocytes from healthy donors (Granulo, n =12) compared with primary APL cells (n =31). The expression levels of the NEAT1 isoforms were evaluated by qRT-PCR. Malat1 lncRNA served as negative control and shown in Additional file 1: Figure S1A. Measured cycle threshold (Ct) values represent log2 expression values. The values were normalized to the expression level of the housekeeping gene *ACTB*. Each data point represents 1 patient sample.

Because NEAT1 has been proposed to control several biological processes, including the stress response [21] and cellular differentiation [15], we therefore initially examined the expression level of NEAT1 in peripheral blood mononuclear cells (PBMCs) from 31 cases with de novo APL (13 males and 18 females with a median age of 28.5 years and a range of 17–52 years) expressing the *PML-RARα* fusion gene, which is characterized by leukemia blasts blocked at the promyelocyte stage of differentiation. NEAT1 lncRNA is comprised of two isoforms, NEAT1_1 (3.7 kb in humans) and NEAT1_2 (23 kb in humans ) (Figure 1A), and we used two primer pairs that were designed as previously described to quantify NEAT1 RNA isoforms by real-time quantitative reverse transcription-PCR (qRT-PCR) [22]. One primer set recognizes both NEAT1_1 and NEAT1_2 (total NEAT1), while the other recognizes only NEAT1_2. qRT-PCR revealed that both NEAT1 and NEAT1_2 were significantly decreased in APL patient samples compared with normal granulocytes (Figure 1B), but not the Malat1 lncRNA (Additional file 1: Figure S1A). This result suggested that NEAT1 may be involved in APL pathogenesis.

### NEAT1 is suppressed by PML-RARα, and ATRA restores NEAT1 expression

**Figure 2:**
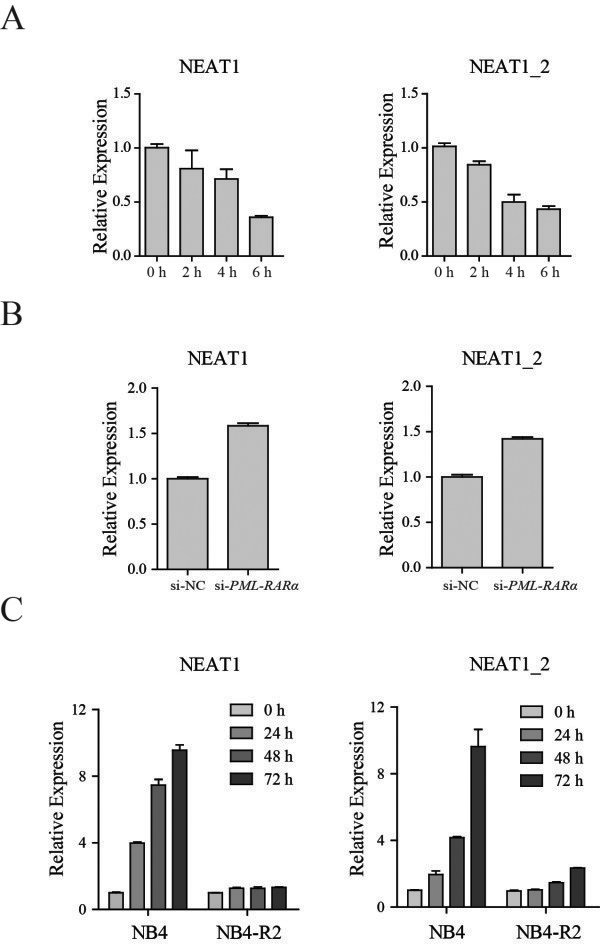
**lncRNA NEAT1 is repressed in cells expressing PML-RARα and upregulated in response to ATRA. (A)** qRT–PCR analysis of NEAT1 in U937-PR9 cells treated with 100 μM ZnSO_4_ at the indicated time points. A time series of induction for the PML-RARα protein by ZnSO_4_ is shown in Additional file 2: Figure S2A. **(B)** qRT–PCR analysis of NEAT1 after knocking down PML-RARα. **(C)** NB4 and NB4-R2 cells were treated with 1 μM ATRA. NEAT1 was measured by qRT-PCR and normalized to the housekeeping gene *ACTB*. The panels show the mean ± SD of a representative experiment performed in triplicate.

The PML-RARα fusion protein is known to be the initiating factor for APL development by transcriptionally repressing retinoic acid and non–retinoic acid target genes, we wondered whether NEAT1 downregulation was a consequence of PML-RARα expression; thus, we investigated the relationship between NEAT1 and PML-RARα. To examine whether the expression of the NEAT1 lncRNAs is changed by PML-RARα expression, we used a U937-derived cell line, U937-PR9, which contains a zinc-inducible PML–RARα. U937-PR9 cells were treated with 100 μM ZnSO_4_ for the indicated time, and PML-RARα was significantly upregulated (Additional file 2: Figure S2A). Using this system, we found that NEAT1 expression was significantly reduced in PML-RARα-expressing cells (Figure 2A), but not in parental U937 cells (Additional file 2: Figure S2B), indicating that NEAT1 downregulation may be a consequence of PML-RARα accumulation. Next, we used a small interfering RNA (siRNA), which was designed according to a previous report in which PML-RARα was effectively knocked down by specifically targeting its breakpoint region [20]. The knockdown of PML-RARα by transfection with *si-PML-RARα* was confirmed by qRT-PCR and western blot (Additional file 2: Figure S2C). As expected, we found that the expression of NEAT1 was increased by PML-RARα knockdown (Figure 2B).Because APL treatment leads to the clearance of leukemia cells and loss of PML-RARα transcripts, we next investigated the expression of NEAT1 and PML-RARα in NB4 cells before and after ATRA treatment (1 μM, stock: 10 mM in EtOH) to further establish the relationship between NEAT1 and PML-RARα. Using qRT-PCR, the expression level of total NEAT1 and NEAT1_2 in NB4 cells were rapidly increased upon treatment with ATRA. Importantly, NEAT1 expression was not significantly changed in ATRA-resistant NB4-R2 cells upon ATRA treatment, excluding the possibility that the NEAT1 induction observed in NB4 cells represents a nonspecific stress response to ATRA treatment rather than being functional in differentiation (Figure 2C).

### NEAT1 inhibition attenuates the myeloid differentiation of APL cells

**Figure 3:**
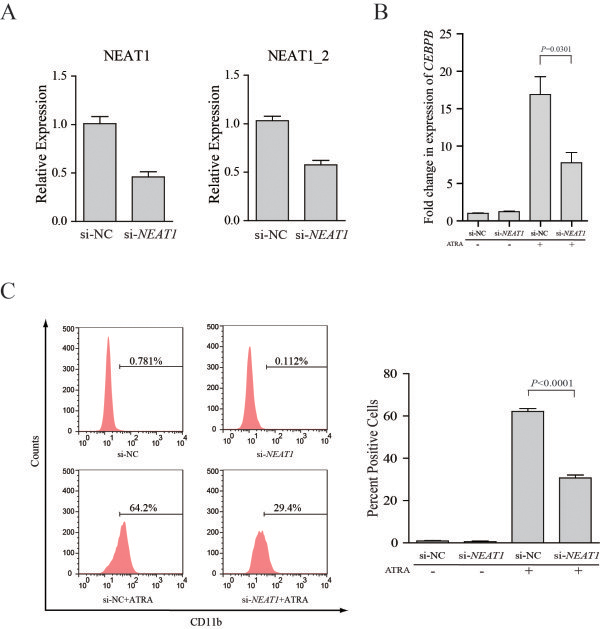
**Knocking down NEAT1 impairs neutrophil differentiation in APL cells. (A)** 48 hrs after transfection, the knockdown efficiency was confirmed by qRT-PCR. **(B)** CEBPB mRNA levels were measured by qRT–PCR and are given as n-fold changes compared with untreated cells and normalized to the housekeeping gene *ACTB*. **(C)** Flow cytometry analysis of ITGAM/CD11b surface expression of control and si-*NEAT1* cells upon 48 h of ATRA-treatment (1 μM). CD11b expression was measured by flow cytometry and values were normalized to untreated control cells. Data are shown as the mean ± SD of three separate experiments.

The above data show that the PML-RARα oncoprotein represses the expression of NEAT1, and ATRA treatment reverts the transcriptional repression mediated by the PML-RARα fusion protein and increases NEAT1, suggesting that NEAT1 may be involved in cell differentiation and leukemogenesis. We then explored the functional role of NEAT1 in the ATRA-induced myeloid differentiation of APL cells. NEAT1 has been previously shown to be effectively knocked down by siRNA [17, 23], and it efficiently attenuated the NEAT1 RNA level in this system compared with control siRNA (Figure 3A). Transduced cells were treated with ATRA, and after 48 h, the expression level of CEBPB mRNA and a membrane antigen (ITGAM/CD11b) associated with granulocytic cell differentiation was measured [24]. We found significantly reduced CEBPB mRNA and ITGAM/CD11b levels in NB4 cells transfected with *si-NEAT1* compared with control cells upon ATRA treatment (Figure 3B and C). These data reveal a novel function for NEAT1 in myeloid differentiation.

## Discussion

In this study, we show that NEAT1 expression is repressed by PML-RARα. In addition, we provide evidence that NEAT1 expression is involved in the differentiation of APL cells.

PML-RARα is a potent transcriptional repressor in APL cells, and it blocks promyelocyte differentiation. An interesting characteristic of this oncogenic protein is that its transcriptional repression effects can be reverted with pharmacologic doses of ATRA, resulting in the reactivation of genes essential for definitive myeloid differentiation [2, 3, 25]. Recently, lncRNAs have been shown to be dysregulated in various cancers, and several lncRNAs have been functionally linked to cancer and cell differentiation [13, 15]. In this study, our findings of particular low NEAT1 levels in APL cells indicate that NEAT1 transcription is further repressed by PML-RARα.Indeed, we found that NEAT1 was repressed by PML-RARα, and this repression could be relieved by ATRA. These results suggest that NEAT1 expression is regulated by PML-RARα. Unlike ATRA, arsenic trioxide induces limited transcriptional changes of NEAT1 in APL cells (Additional file 3: Figure S3A). This result may due to arsenic trioxide induces significant cell death but only very limited differentiation [26, 27], and upregulation of NEAT1 in ATRA-treated APL cells is a consequence of the relief of PML-RARα-mediated transcriptional repression. Notably, previous studies have indicated PML-RARα is able to interact with many other transcription factors, such as PU.1, providing the potential for the oncoprotein to target genes primarily regulated by other transcription factors [7, 28]. However, due to the structural and functional complexity of PML-RARα, whether this regulation is by direct DNA binding or by other transcription factors requires further investigation. More importantly, this lncRNA is involved in the differentiation of APL cells, suggesting an interesting characteristic for the PML-RARα oncogenic protein in the regulation of gene expression by NEAT1. The mammalian nucleus is highly organized and contains several membraneless subcompartment nuclear bodies, including nucleoli, paraspeckles, PML bodies and speckles, which are thought to be involved in gene regulation [29]. NEAT1 is a critical component of the paraspeckle structure, and paraspeckles have been proposed to be involved in the regulation of gene expression through the control of the nuclear retention of mRNAs containing long inverted repeats, which are capable of forming intramolecular double-stranded RNAs subject to adenosine-to-inosine editing [17]. More recently, NEAT1 was also shown to be involved in transcriptional regulation by sequestrating a transcriptional regulator [21]. Previous studies have demonstrated that PML-RARα was capable of promoting leukemic transformation by impairing the formation of functional PML nuclear bodies [30]. Thus, PML-RARα may influence the nuclear retention of structured mRNAs or gene transcription, which is indispensable for myeloid differentiation, through NEAT1. This finding is the first evidence that a lncRNA cooperates with a fusion protein and plays a critical role in the response to treatment. However, the underlying molecular mechanisms of NEAT1 and potential roles of paraspeckles in APL require further investigation.

In conclusion, we report abnormally decreased expression of the nuclear long noncoding RNA NEAT1, which is responsible for the differentiation block in blast cells in APL. These findings provide a more comprehensive understanding of APL pathogenesis. Because NEAT1 plays an important role in the posttranscriptional and transcriptional regulation of gene expression and we found that the expression of this nuclear long non-coding RNA was regulated by PML-RARα, it remains an interesting open question whether subsets of genes essential for myeloid differentiation are also modulated by perturbation of NEAT1 expression.

## Conclusions

Taken together, these results are the first to assign a biological function to the nuclear long noncoding RNA NEAT1 in the myeloid differentiation of APL cells and may lead to a fuller understanding of the molecular events leading to APL.

## Electronic supplementary material

Additional file 1: Figure S1: Analysis of Malat1 expression in APL primary patient samples. (A) qRT–PCR and western blot analysis of Malat1 expression in granulocytes from healthy donors (Granulo, n =12) compared with primary APL cells (n =31). Measured cycle threshold (Ct) values represent log2 expression values. The values were normalized to the expression level of the housekeeping gene *ACTB*. Each data point represents 1 patient sample. (DOCX 890 KB)

Additional file 2: Figure S2: NEAT1 is suppressed by PML-RARα. (A) qRT–PCR and western blot analysis of PML-RARα in U937-PR9 cells induced with 100 μM ZnSO_4_. (B) qRT–PCR analysis of NEAT1 in U937 cells treated with 100 μM ZnSO_4_ at the indicated time points. NEAT1 was normalized to the housekeeping gene *ACTB*. The panels show the mean ± SD of a representative experiment performed in triplicate. (C) qRT–PCR analysis and western blots were performed to detect PML-RARα in NB4 cells after transfection with *si-PML-RARα* (Student’s t test was used to calculate the p value). (DOCX 2 MB)

Additional file 3: Figure S3: Analysis of NEAT1 expression. (A) qRT–PCR analysis of NEAT1 in NB4 cells treated with 2 μM arsenic trioxide at the indicated time points. The *ACTB* level is shown as a loading control. (DOCX 883 KB)
