# Supplementary figures and images for: Inhibition of long non-coding RNA NEAT1 impairs myeloid differentiation in acute promyelocytic leukemia cells
Source: BMC Cancer. 2014 Sep 23;14:693. doi: 10.1186/1471-2407-14-693 (PMC4180842; doi:10.1186/1471-2407-14-693)

**Additional file 1: Figure S1.**


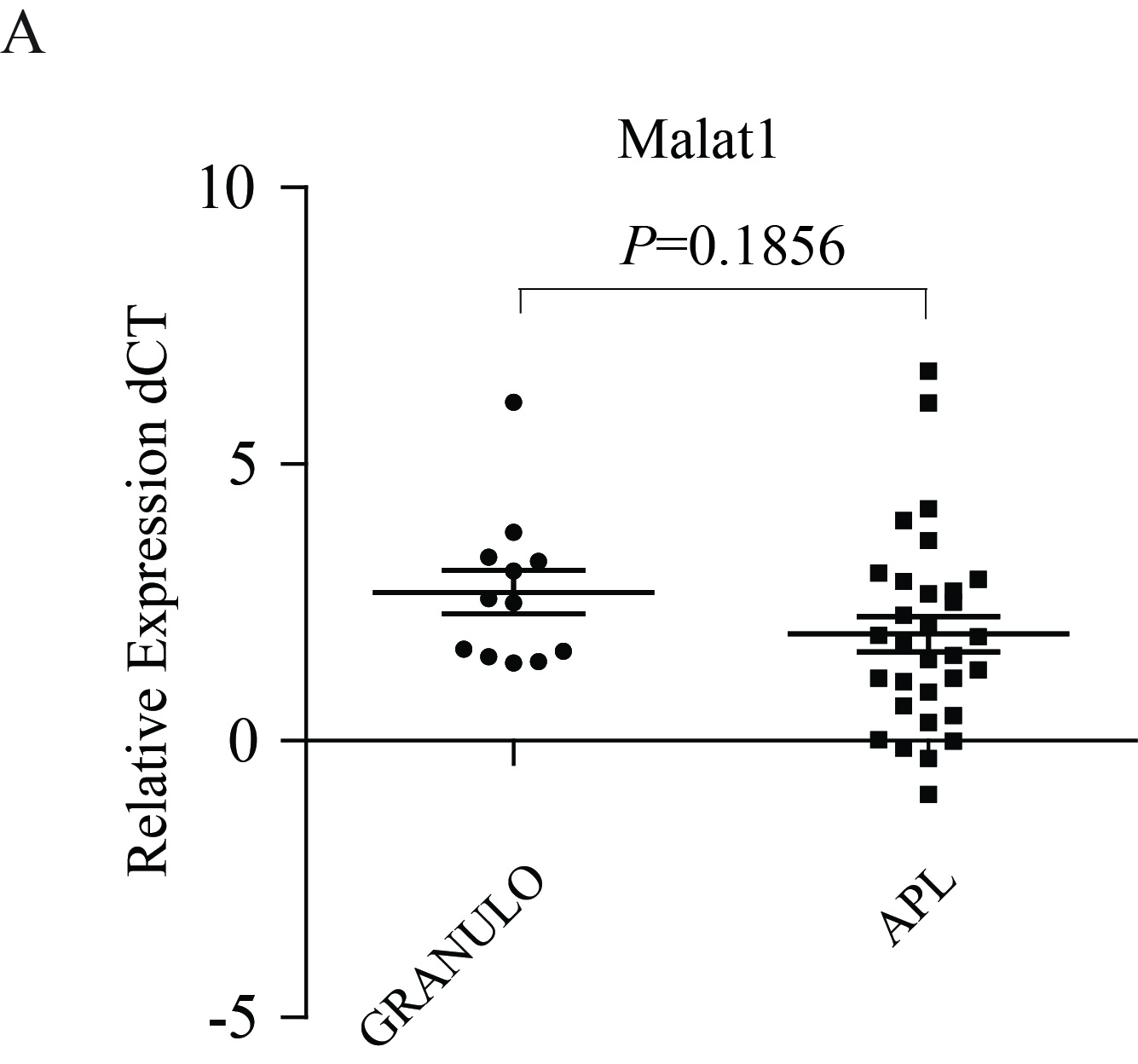

Supplement: Supplementary file 1 — Additional file 1: Figure S1: Analysis of Malat1 expression in APL primary patient samples. (A) qRT–PCR and western blot analysis of Malat1 expression in granulocytes from healthy donors (Granulo, n =12) compared with primary APL cells (n =31). Measured cycle threshold (Ct) values represent log2 expression values. The values were normalized to the expression level of the housekeeping gene ACTB. Each data point represents 1 patient sample. (DOCX 890 KB) [file 12885_2014_4869_MOESM1_ESM.docx]

**Additional file 2: Figure S2.**


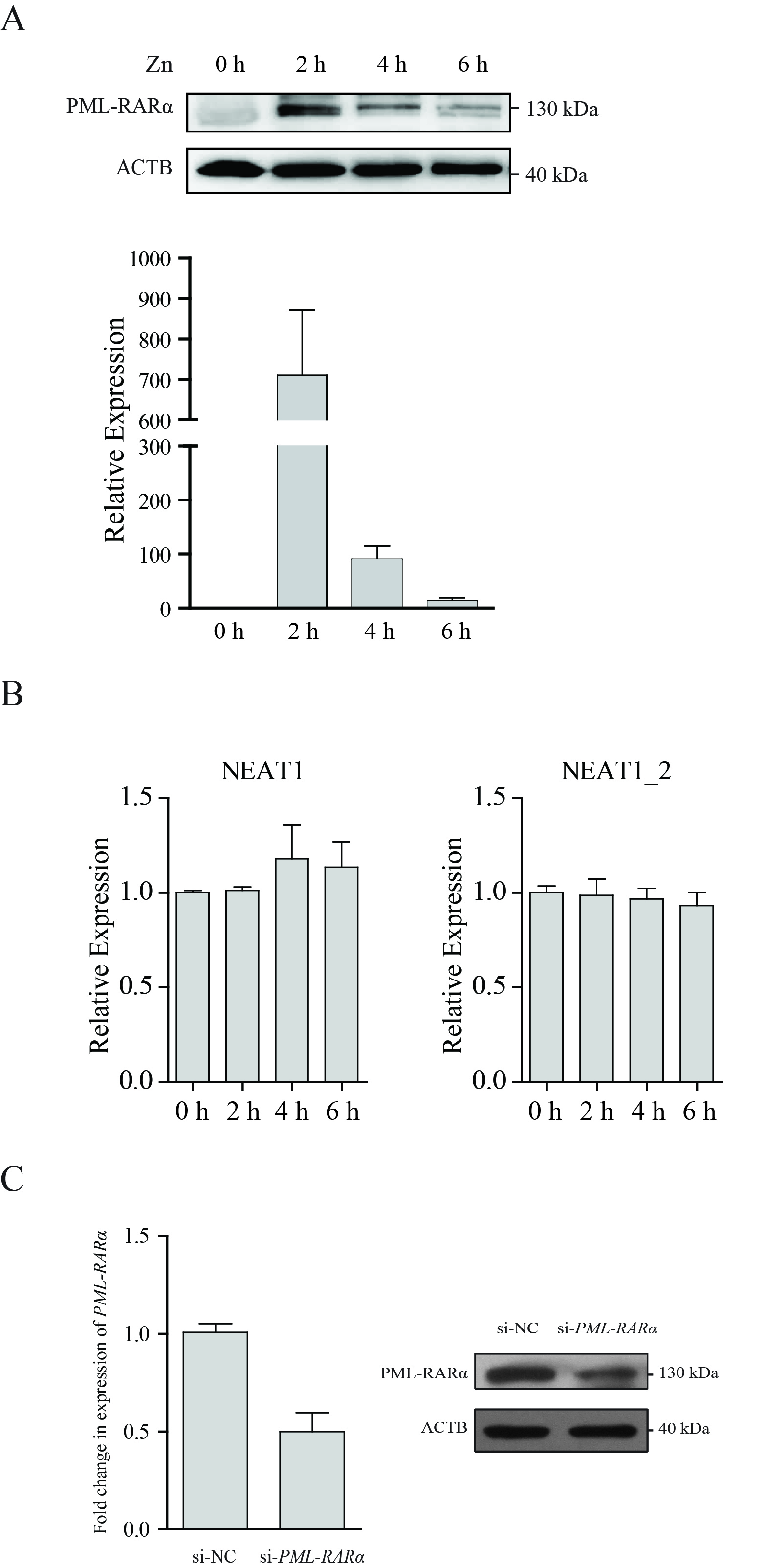

Supplement: Supplementary file 2 — Additional file 2: Figure S2: NEAT1 is suppressed by PML-RARα. (A) qRT–PCR and western blot analysis of PML-RARα in U937-PR9 cells induced with 100 μM ZnSO4. (B) qRT–PCR analysis of NEAT1 in U937 cells treated with 100 μM ZnSO4 at the indicated time points. NEAT1 was normalized to the housekeeping gene ACTB. The panels show the mean ± SD of a representative experiment performed in triplicate. (C) qRT–PCR analysis and western blots were performed to detect PML-RARα in NB4 cells after transfection with si-PML-RARα (Student’s t test was used to calculate the p value). (DOCX 2 MB) [file 12885_2014_4869_MOESM2_ESM.docx]

**Additional file 3: Figure S3.**


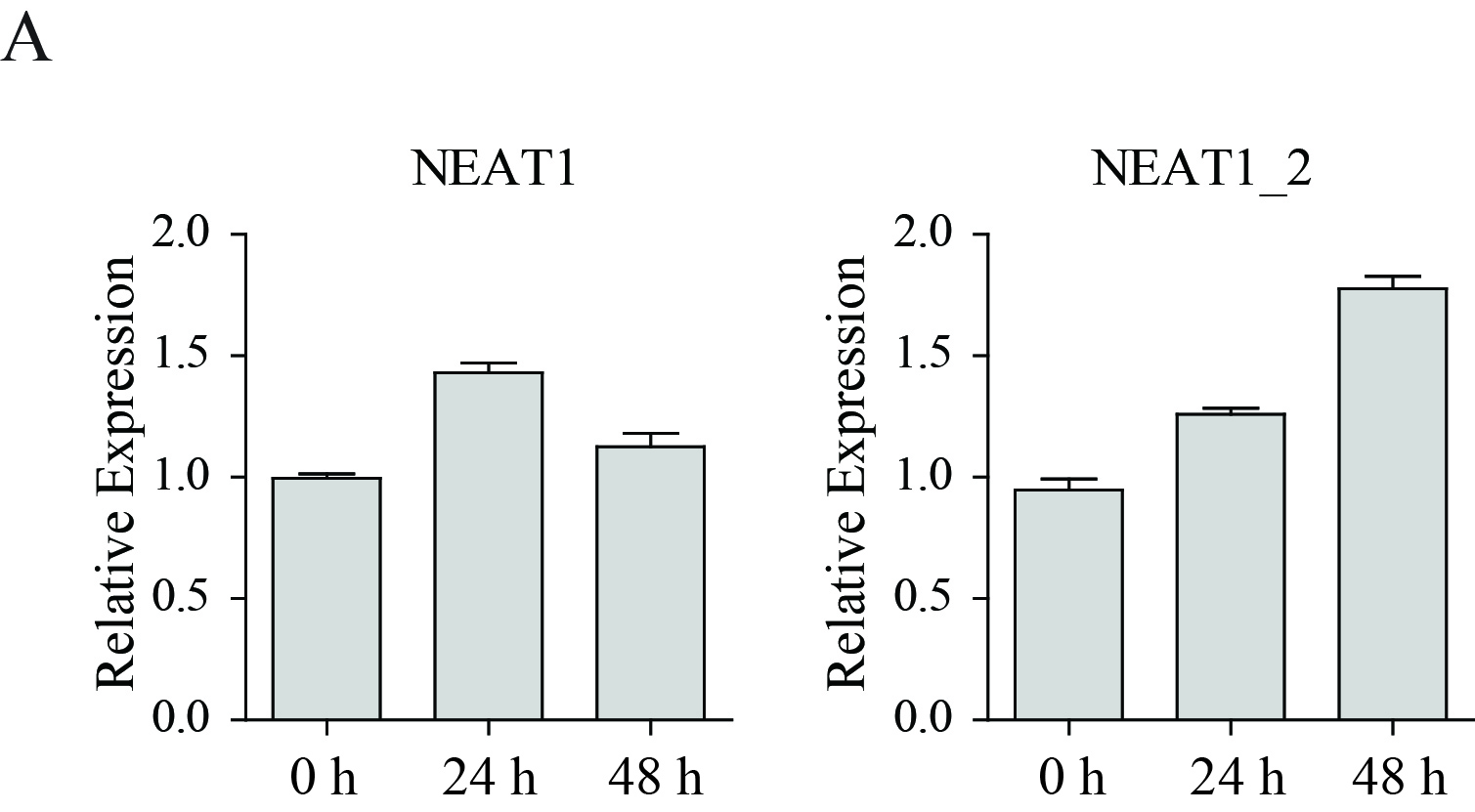

Supplement: Supplementary file 3 — Additional file 3: Figure S3: Analysis of NEAT1 expression. (A) qRT–PCR analysis of NEAT1 in NB4 cells treated with 2 μM arsenic trioxide at the indicated time points. The ACTB level is shown as a loading control. (DOCX 883 KB) [file 12885_2014_4869_MOESM3_ESM.docx]
